# The effect of the modified fat-protein unit algorithm compared with that of carbohydrate counting on postprandial glucose in adults with type-1 diabetes when consuming meals with differing macronutrient compositions: a randomized crossover trial

**DOI:** 10.1186/s12986-023-00757-w

**Published:** 2023-10-16

**Authors:** Yunying Cai, Mengge Li, Lun Zhang, Jie Zhang, Heng Su

**Affiliations:** 1 The Endocrinology Department, First People’s Hospital of Yunnan Province, The Affiliated Hospital of Kunming University of Science and Technology, Kunming, 650032 China; 2 Wenjiang District People’s Hospital of Chengdu, Chengdu, 611130 China; 3 The Clinical Nutrition Department, First People’s Hospital of Yunnan Province, The Affiliated Hospital of Kunming University of Science and Technology, Kunming, 650032 China

**Keywords:** Carbohydrate counting, Modified fat-protein unit algorithm, High protein-fat meal, Type − 1 diabetes

## Abstract

**Background:**

The optimization of glucose control in type-1 diabetes is challenged by postprandial glycemic variability. This study aimed to compare the postprandial glycemic effects of carbohydrate counting and the modified fat-protein unit (FPU) algorithms following meals with different protein and fat emphases in adults with type-1 diabetes.

**Methods:**

Thirty adults with type-1 diabetes aged 18 to 45 years participated in a randomized crossover trial. In a random order, participants consumed four test meals with equivalent energy and different macronutrient emphases on four separate mornings. The modified FPU algorithms and carbohydrate counting were used to determine the insulin dose for the test meals. A continuous glucose monitoring system was used to measured postprandial glycemia.

**Results:**

Compared with carbohydrate counting, the modified FPU algorithm significantly decreased the late postprandial mean glucose levels (p = 0.026) in high protein-fat meals. The number of hypoglycemia episodes was similar between insulin dosing algorithms for the high protein-fat meals; hypoglycemic events were considerably higher for the modified FPU in the normal protein-fat meal (p = 0.042).

**Conclusions:**

The modified FPU algorithm may improve postprandial glycemic control after consuming high protein-fat meals in adults with type-1 diabetes but may result in increased hypoglycemia risk when used with a normal protein-fat meal.

## Introduction

Postprandial glucose fluctuations are a significant contributor to the challenge of glycemic control in type-1 diabetes. According to the International Society for Pediatric and Adolescent Diabetes (ISPAD) and the American Diabetes Association (ADA), carbohydrate counting (CC) is the standard prandial insulin dose estimation method [1, 2]. However, dietary fat and protein have been demonstrated to contribute to postprandial glycemic excursions [3–6], as high fat or protein results in a delayed and protracted rise in postprandial glycemia between 1.5 and 6 h after the meal [3–6]. In contrast, all of those studies indicated that an additional bolus of insulin is necessary for high-fat or high-protein diets, with no agreement on how to calculate the effects of dietary fat or protein.

Recently, novel algorithms for assessing the glycemic impact of fat and protein have been presented. For instance, the Pankowska equation defines a ‘fat-protein unit (FPU)’ as 100 kcal of fat or protein, which needs the same quantity of insulin as 10 g of carbohydrates [7]. Nevertheless, a more recent study discovered a decreased demand for insulin for protein, approximately 200 kcal equaling 10 g of carbohydrates [2, 8]. Currently, no clinical studies have compared the acute postprandial glycemic effects of CC and FPU for meals with different fat and protein contents in Chinese adults with type-1 diabetes.

This study aimed to compare the impact of the modified FPU to the conventional CC on postprandial glucose excursions following normal and high protein-fat meals. It was anticipated that when the modified FPU instead of CC was used to calculate mealtime insulin dosing, the 5-h postprandial glucose excursions would be reduced.

## Materials and methods

At the First People’s Hospital of Yunnan Province, a randomized, open-label, inside-subject crossover trial was conducted. Before enrolling in the trial, each patient aged 18 years or older submitted written informed consent. The study was registered with the Chinese Clinical Trial Registry (ChiCTR2100049763) and endorsed by the First People’s Hospital Medical Ethics Committee of Yunnan Province.

### Participants

Patients between 18 and 45 who had been diagnosed with T1D for more than a year and received multiple insulin injection (MDI) therapy were included. Furthermore, standard body mass index (BMI), normal thyroid function, and recent hemoglobin A1c (HbA1c) levels ranged from 6.5 to 9% before recruitment was needed.

The exclusion criteria were as follows: (i) any comorbid illnesses, such as coeliac disease and autoimmune thyroid disease; (ii) fasting blood glucose on trial day greater than 10 mmol/L or lower than 3.9 mmol/L; (iii) using corticosteroid or other drugs that might impair gastric emptying; (v) having any dietary restrictions, such as food allergies; (vi) having ketoacidosis within 24 h of consuming the test meals.

### Study protocol

The FreeStyle Libre Flash Glucose Monitoring System (FGM, Abbott) was implanted in the subcutaneous tissue of the abdomen area or upper buttocks on the first trial day. The insertion was placed one day before the first meal to avoid bias. All patients were educated and trained on how to handle their FGMS daily.

Patients randomly consumed four test meals (NPM-a, NPM-b, HPFM-a, and HPFM-b) on four different occasions, each separated by three days. NPM-a was a normal protein meal based on CC; NPM-b was a normal protein meal based on the modified FPU method; HPFM-a was a high protein-fat meal based on CC; and HPFM-b was a high protein-fat meal based on the modified FPU method. Each meal was prepared at 07:00 AM and made in the hospital kitchen.

Because the influence of the first meal on glucose levels lasts longer depending on the content of the meal, the breakfast meal was chosen as the test meal to eliminate any confounding second-meal effect. Participants were advised to refrain from vigorous exercise and high-fat, high-protein meals the day before the test meal and to fast for 10 h before the test meal. Controlled circumstances were used throughout the trial, and glycemic response factors were minimized. Each meal was consumed within thirty minutes; no food or drink was permitted throughout the 5-hour postprandial period unless symptomatic hypoglycemia occurred.

NPM contains the following ingredients: [milk (250 mL), egg (50 g), beef (50 g), whole wheat bread (75 g); 53 g carbohydrate, 32 g protein, 17 g fat], HPFM contains the following ingredients: [milk (250 mL), egg (50 g), beef (150 g), whole wheat bread (37.5 g);35 g of carbohydrate, 49 g of protein, 18.5 g of fat]. The insulin dosages for NPM-a and HPFM-a were estimated based on the carbohydrate content of the meals; for NPM-b and HPFM-b, the modified FPU was used to determine the insulin dose. The composition of NPM and HPFM meals and the total insulin dosage for each meal are detailed in Table 1.

**Table 1:** Composition of test meals

Parameters	Unit	NPM-a	NPM-b	HPFM-a	HPFM-b
Energy	kcal	505	505	505	505
Carbohydrate	g	53	53	35	35
	%	50.8	50.8	27.3	27.3
Protein	g	32	32	49	49
	%	22.4	22.4	38.5	38.5
Fat	g	17	17	18.5	18.5
	%	25.3	25.3	32.8	32.8
Fiber	g	0.05	0.05	0.04	0.04
	%	0	0	0	0
Total insulin administration [mean (SD)]	IU	6.97 ± 0.61	8.70 ± 0.69	4.92 ± 0.54	7.23 ± 0.68

NPM-a, a normal protein meal based on CC; NPM-b, a normal protein meal based on the modified FPU method; HPFM-a, a high protein-fat meal based on CC; HPFM-b, a high protein-fat meal based on the modified FPU method

Before the test meal, the FGMS was tested for proper performance and adherence to the study protocol. For patients receiving MDI therapy, a short-acting insulin bolus was injected subcutaneously at the start of each meal.

All patients utilized the insulin–carbohydrate ratio for mealtime boluses for CC meals (NPM-a, HPFM-a). The current ICR was determined by dividing the total daily insulin dosage by 500 and remained constant throughout the control and test meals. The CC algorithms did not account for fat or protein.

The insulin-to-fat-protein ratio and ICR were employed to administer mealtime boluses for the modified FPU counting meal (NPM-b, HPFM-b). The modified FPU is defined as one FPU was 200 kcal of fat or protein that requires the same quantity of insulin as 10 g of carbohydrates. The mealtime insulin dose was determined and delivered depending on the meal’s carbohydrate, lipid, and protein content. For the NPFM meal, the protein and fat content was one FPU, and HPFM was two FPU.

The test was terminated when hypoglycemia was detected using capillary blood glucose and was repeated the next day. Patients suffering hypoglycemia (glucose levels less than 3.9 mmol/L) were instructed to take juice containing 15 g of carbohydrates. During the research, no patient experienced severe hypoglycemia.

### Measurements

FGM was used to monitor interstitial fluid glucose levels, and only the 5-hour postprandial period FGMS data were utilized for analysis. Aside from FGMS data, capillary blood glucose levels were assessed using the Abbott blood glucose monitoring system at the beginning of the meals (T = 0), 120 min after meals, and when symptomatic hypoglycemia occurred.

FGMS measurements yielded the following outcome parameters: (1) mean glucose levels, which were recorded every 15 min with the FGMS; (2) peak glucose level, which was the highest level recorded during the 5-h postprandial period; the time of its occurrence was used to determine the time to peak glucose; (3) incremental area under the glucose excursion curve, which was determined as the area under the glucose curve during the 5-hour postprandial period with the glucose level at T = 0 as the baseline; (4) hypoglycemic episodes, which were defined as glucose levels less than 3.9 mmol/L measured by FGMS, at which time the onset of hypoglycemia was recorded; (5) time above range (> 10 mmol/L) during the 5-hour postprandial period; and (6) glucose excursions, which were defined as variations in glucose levels measured every thirty minutes.

### Statistical analysis

The study size was determined by a prospective clinically significant difference in mean glucose level of 2.5 mmol/L and a 2 mmol/L within-subject SD in glucose levels when using FGMS. The predicted study size was 16 based on a power of 80% and a two-sided significance level of 5%.

Baseline data for categorical variables are presented as counts and percentages, while the mean and SD are used to represent continuous variables with normal distribution and the median and interquartile range (IQR) are used for nonnormal continuous variables.

SPSS (25.0, IBM Corp., Armonk, NY, USA) was used to perform all statistical analyses. The Mann–Whitney U test or t test was used to compare continuous variables. One-way repeated-measures analysis of variance was used to compare test meals. Generalized linear mixed models accounted for repeated measurements within the same individual, such as glucose levels, excursions, and time above range.

## Results

Thirty-six T1D patients receiving basal-bolus insulin were recruited for the study. Six were eliminated due to the inability to finish the study (2/36) or hypoglycemia (4/36), leaving 30 participants for analysis. Four males and 26 females had a median age of 32 years (range 18–45 years) and a median duration of diabetes of 13 years (range 2–35 years). The mean body mass index (BMI) was 21.2 ± 1.6 kg/m2, the mean glycated hemoglobin (HbA1c) was 7.1 ± 1.29%, the mean ICR was 1 unit per 15.74 ± 4.75 g carbohydrate, and the mean total daily insulin dose was 0.65 ± 0.19 units/kg/day. Table 1 shows meal-type mean insulin dosages. Compared with that of CC, the mean insulin dose for the modified FPU was 47% higher for a HPFM and 25% higher for a NPM.

### Peak glucose levels

Table 2 shows peak glucose levels and time to peak for each test meal. In response to the NPM, the FPU algorithm significantly lowered the mean peak glucose (11.87 ± 0.74 vs. 9.33 ± 0.59 mmol/L, *p* = 0.012) without altering the time to peak glucose level. CC and FPU had similar peak glucose levels after the HPFM. However, the FPU algorithm changed the mean time to peak glucose (242.31 ± 21.20 vs. 153.46 ± 25.54 min, *p* = 0.008).

### Mean glucose levels

The FPU algorithm reduced mean glucose levels (240–300 min) after each test meal compared to CC (Table 2).

**Table 2:** Glycemic outcome parameters

	NPM-a	NPM-b	HPFM-a	HPFM-b	*p* value NPM	*p* value HPFM
Fasting glucose(mmol/L)	6.67 ± 0.47	6.71 ± 0.52	6.47 ± 0.40	6.16 ± 0.27	0.942	0.448
Peak glucose(mmol/L)	11.87 ± 0.74	9.33 ± 0.59	10.01 ± 0.51	9.40 ± 0.61	0.012	0.17
Time to peak glucose (min)	147.00 ± 16.38	139.00 ± 24.91	242.31 ± 21.20	153.46 ± 25.54	0.79	0.008
Mean glucose levels (mmol/L) (0-120 min)	8.98 ± 0.56	6.99 ± 0.47	7.10 ± 0.40	6.79 ± 0.33	0.008	0.501
Mean glucose levels (mmol/L) (0-240 min)	9.41 ± 0.64	6.96 ± 0.38	7.71 ± 0.38	7.23 ± 0.38	0.003	0.179
Mean glucose levels (mmol/L) (0-300 min)	9.25 ± 0.62	6.96 ± 0.34	7.94 ± 0.39	7.34 ± 0.41	0.003	0.07
Mean glucose levels (mmol/L) (120-240 min)	9.94 ± 0.77	6.94 ± 0.48	8.34 ± 0.40	7.66 ± 0.53	0.002	0.142
Mean glucose levels (mmol/L) (240–300 min)	8.67 ± 0.70	6.97 ± 0.47	8.91 ± 0.52	7.82 ± 0.69	0.038	0.026
Area under the curve(mmol/l/min)	2866.80 ± 192.51	2091.07 ± 105.63	2360.69 ± 110.59	2207.23 ± 123.95	0.001	0.166
Number of hypoglycemic events	0	5(33.3)	1(7.69)	1(7.69)	0.042	1
Time to onset of hypoglycemic events (min)	0	123.00 ± 37.17	45.00 ± 0.00	60.00 ± 0.00		0

NPM-a, a normal protein meal based on CC; NPM-b, a normal protein meal based on the modified FPU method; HPFM-a, a high protein-fat meal based on CC; HPFM-b, a high protein-fat meal based on the modified FPU method

### Incremental area under the glucose excursion curve

**Fig. 1:**
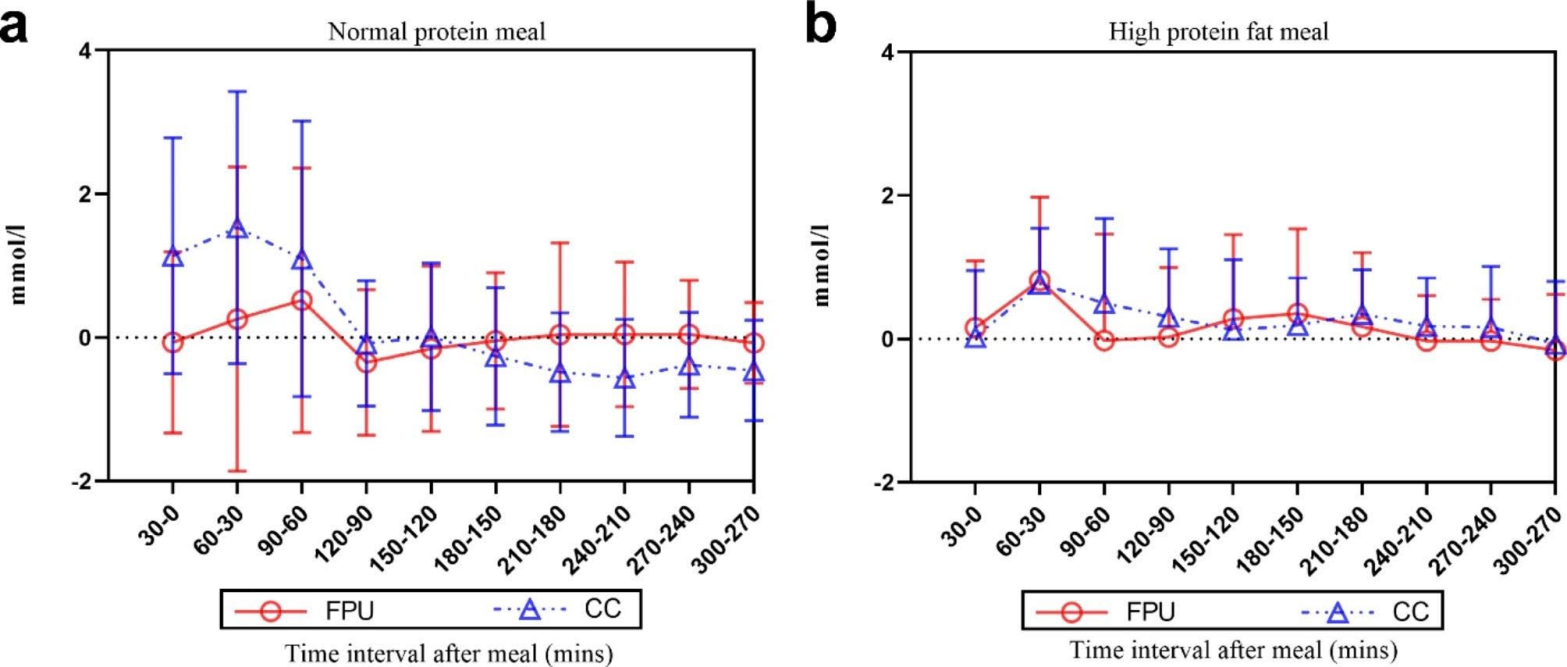
Glucose excursions (mmol/L) at 30-minute intervals by continuous glucose monitoring system (CGMS) (x ®±s); (**a**) normal protein meal; (**b**) high protein-fat meal

In response to the NPM, the 5-hr iAUC for the FPU algorithm was substantially less than that for CC. The iAUC did not differ substantially between CC and the FPU algorithm in the HPFM meal (Table 3). Glucose excursions (mmol/L) at 30-minute intervals are displayed in Fig. 1.

**Table 3:** Indices of glycemic variability for test meals in 300 min (mean values and standard errors (SE), n = 30)

	NPM-a	NPM-b	HPFM-a	HPFM-b	*p* value NPM	*p* value HPFM
iAUC_0 − 30_	173.93 ± 19.27	117.16 ± 33.57	79.80 ± 26.40	123.41 ± 25.77	0.076	0.136
iAUC_0 − 60_	387.08 ± 48.34	222.81 ± 55.28	191.95 ± 53.17	281.73 ± 52.72	0.009	0.086
iAUC_0 − 90_	673.00 ± 67.06	346.86 ± 73.22	358.00 ± 76.11	444.08 ± 76.56	0	0.239
iAUC_0 − 120_	941.43 ± 97.38	463.95 ± 95.44	540.99 ± 94.32	605.08 ± 89.87	0	0.423
iAUC_0 − 150_	1216.66 ± 132.71	584.33 ± 123.47	743.32 ± 111.99	786.78 ± 105.60	0.001	0.614
iAUC_0 − 180_	1490.96 ± 168.40	700.71 ± 151.49	980.72 ± 118.91	993.13 ± 118.78	0.001	0.895
iAUC_0 − 210_	1757.61 ± 201.06	825.33 ± 173.67	1234.49 ± 125.93	1193.68 ± 143.25	0.001	0.696
iAUC_0 − 240_	2023.61 ± 226.07	971.93 ± 191.84	1485.59 ± 133.54	1404.08 ± 171.21	0.001	0.44
iAUC_0 − 270_	2276.46 ± 248.54	1128.09 ± 210.08	1762.57 ± 148.23	1602.12 ± 200.92	0.001	0.249
iAUC_0 − 300_	2521.63 ± 268.32	1275.13 ± 230.87	2031.22 ± 163.24	1788.71 ± 227.06	0.001	0.137
MAGE	5.07 ± 0.79	3.92 ± 0.61	3.23 ± 0.51	3.24 ± 0.59	0.259	0.989
∆Peak(mmol/L)	5.25 ± 0.74	2.62 ± 0.51	3.52 ± 0.45	3.22 ± 0.64	0.006	0.508
∆Low(mmol/L)	-0.65 ± 0.35	-2.21 ± 0.62	-0.62 ± 0.18	-0.75 ± 0.25	0.005	0.539

NPM-a, a normal protein meal based on CC; NPM-b, a normal protein meal based on the modified FPU method; HPFM-a, a high protein-fat meal based on CC; HPFM-b, a high protein-fat meal based on the modified FPU method. iAUC: incremental area under the glucose excursion curve; MAGE: mean amplitude of glycemic excursions

### Time in range (3.9–10 mmol/L)

The time in range (3.9–10 mmol/l) for 300 min following each test meal is displayed in Table 4. In the NPM, participants spent less time within the target range when using CC (65.74 ± 8.92%) than when using FPU (85.43 ± 3.90%). Following the HPFM meal, the FPU algorithm and CC show a similar percentage of time spent within the target range.

**Table 4:** Glycaemic outcomes by algorithm following different test meals

		Meantime in target Range (3.9–10 mmol/L) (%)		Meantime above target Range(> 10 mmol/L) (%)		Meantime below target range (< 3.9 mmol/ L) (%)	
Algorithm		Meal type		Meal type		Meal type	
		NPM	HPFM	NPM	HPFM	NPM	HPFM
	CC	65.74 ± 8.92	87.27 ± 6.48	41.17 ± 9.10	14.49 ± 7.82	0	0.64 ± 0.64
	FPU	85.43 ± 3.90	88.94 ± 5.08	11.50 ± 4.04*	9.42 ± 5.00	3.78 ± 1.68*	1.54 ± 1.54

CC, carbohydrate counting; FPU, fat-protein unit; NPM: normal protein meal; HPFM: high protein-fat meal.* p < 0.05 for FPU vs. CC

### Time above range(> 10 mmol/L)

The time above range associated with the use of the FPU algorithm was substantially less than that associated with the use of CC after each test meal, but marked differences were only identified in the NPMs (Table 4).

### Hypoglycemic episodes

Table 4 shows hypoglycemia episodes. The hypoglycemic events were significantly fewer for CC with the modified FPU in the NPM (0 vs. 3.78 ± 1.68%, *p* < 0.05). In the HPFM meal, there was no significant difference in the number of hypoglycemia incidents between the two dosing regimens.

## Discussion

Our work is the first to prove that additional insulin based on a modified FPU algorithm (200 kcal protein or fat equaling 10 g carbohydrates) can be safely administered before a high-fat, high-protein meal in adults with type-1 diabetes using MDI therapy without raising the risk of hypoglycemia. However, utilizing the modified FPU method instead of CC after a NPM within less than one FPU (200 kcal) was associated with a considerably higher risk of hypoglycemia (approximately 33%).

The majority of guidelines suggest using CC to calculate mealtime insulin dosage for type-1 diabetes. Although all studies have indicated that extra bolus insulin is required for high-fat and high-protein diets, there is no agreement on when and how to estimate the impacts of dietary fat and protein. In this setting, there is no standard insulin therapy algorithm.

The food insulin index (FII) and the Pankowska Equation (FPU) are two novel algorithms that consider protein and fat glycemic impact [9–11]. Studies comparing the effects of FII versus CC on postprandial glycemic responses after consumption of a high protein and fat meal reported that FII had no advantage and was associated with a higher rate of hypoglycemic attacks (approximately 50%) [9–11]. Most research using the Pankowska Eq. (100 kcal equals 10 g of carbohydrates) has shown positive outcomes in reducing postprandial glycemic levels compared to standard CC but at the expense of an increased rate of hypoglycemia [11–14]. Previous studies found that the postprandial hypoglycemia incidence was 35.7–50% for the Pankowska Eqs. [12, 13]. A recent study suggested that there is a reduced need for insulin when accounting for protein and fat, and considering approximately 200 kcal from protein and fat to equal 10 g of carbohydrates may be an acceptable strategy [2]. This recommended dose has yet to be verified in clinical trials.

In the current study, according to the modified FPU algorithm, insulin was dosed approximately 47% higher for a HPFM and 25% higher for a NPM than for CC. The increased insulin dose for both NPM and HPFM meals resulted in a considerably decreased blood glucose excursion for 240–300 min. Compared with CC, the FPU algorithm reduced mean glucose levels (mmol/L) by 1.1 mmol/L (240–300 min) in each test meal. There was no difference in hypoglycemic attacks when the modified FPU algorithm was compared to CC for HPFM meals. This is consistent with what Smith TA et al. reported in children and adolescents with type-1 diabetes: an extra 40% of the insulin dose for CSII insulin and an additional 25% bolus for MDI insulin for a high-fat, high-protein (HFHP) breakfast optimizes postprandial glycemia without a statistically significant increase in hypoglycemia [15, 16]. The modified FPU algorithm reduced postprandial hyperglycemia (0-300 min) and glucose excursion (0-300 min) in NPMs; nevertheless, the modified FPU algorithm significantly increased hypoglycemia within 120 min of a meal. Our research found that meals with varied compositions may necessitate a different insulin dosing strategy; for HFHP meals, a 25–50% increase in the insulin requirement is safe and effective [14–17].

The dietary fat and/or protein threshold that should be calculated for preprandial insulin dosing is still debated. Paterson, MA et al. stated that a glycemic effect was not seen when protein was consumed alone until ≥ 75 g [8]. However, ≥ 12.5 g of protein affected postprandial glucose in a carbohydrate-containing meal. The glucose-raising effect of protein occurred in the late postprandial period and ranged from 90 to 300 min [6]. Schweitzer et al. proposed that a protein unit (50 g protein) equaling one carbohydrate unit (10 g of carbohydrates) was needed, and the author argued that fat should not be considered [18]. Contradicting findings have shown that dietary fat increases glucose levels [19, 20]. Wolpert HA et al. found that fifty grams of fat could double the demand for insulin [20]. Van der Hoogt discovered that compared to low-fat (7 g in a test meal) and low-protein meals (10.6 g in a test meal), eight times more postprandial correction insulin is needed in high-fat (15 g in a test meal) and high-protein (26 g in a test meal) meals [19]. Our study demonstrates that increasing protein and fat content from one FPU to two FPUs without changing total calories reduced the early postprandial glycemic response (0-240 min) in a carbohydrate-containing meal. However, it increased the postprandial blood glucose level (240–300 min). Even in optimal preprandial settings, meal macronutrient composition might cause varied glucose dynamic responses.

According to studies in adults with T1DM, calculating the mealtime insulin dosage depending on the carbohydrate and protein content may be superior to calculations based simply on the carbohydrate content when the energy provided by protein and fat accounted for 80% of the energy in the meal [21, 22]. The current study found that the time above range was reduced from 11.5 to 9.4% with the modified FPU algorithm compared with CC when there was one FPU variation (200 kcal) in the food without changing total calories. Compared to simple CC, the modified FPU algorithm decreased late postprandial (240–300 min) glucose levels. No improvement was found in early and total postprandial glucose levels because each insulin dosing algorithm achieved an excellent time in the glycemic target range; the mean proportion of TIR was 85.4% for CC and 88.94% for the modified FPU algorithm.

This is the first trial comparing modified FPU counting with CC in the context of Chinese dietary patterns. The strengths of this trial include a high retention rate of study participants. The primary limitation of our study is that the observation period was only 5 h after the meal; thus, we cannot determine if the observed benefit with the modified FPU counting would be sustained throughout an extended period. Most of the subjects in the trial had good glycemic control, which might not represent the overall type-1 diabetes population.

## Conclusions

Our findings highlight that the determination of the optimal insulin bolus needs to be individualized, and the dietary macronutrient composition of the meal should be considered. Compared with CC, modified FPU counting could result in reduced late postprandial glycemic excursions and a reduced percentage of time above range when the amount of fat and protein in a meal exceeds two FPU (400 kcal), but FPU had an increased hypoglycemia risk when used for a NPM (less than one FPU). Simple CC is appropriate for NPMs.

## Data Availability

The data supporting this study’s findings are available from the corresponding author upon reasonable request.
